# Aryl hydrocarbon receptor (AhR) reveals evidence of antagonistic pleiotropy in the regulation of the aging process

**DOI:** 10.1007/s00018-022-04520-x

**Published:** 2022-08-20

**Authors:** Antero Salminen

**Affiliations:** Department of Neurology, Institute of Clinical Medicine, University of Eastern Finland, P.O. Box 1627, 70211 Kuopio, Finland

**Keywords:** Immunosuppression, Kynurenine, Lifespan, Longevity, RelB, Retrotransposon

## Abstract

The antagonistic pleiotropy hypothesis is a well-known evolutionary theory to explain the aging process. It proposes that while a particular gene may possess beneficial effects during development, it can exert deleterious properties in the aging process. The aryl hydrocarbon receptor (AhR) has a significant role during embryogenesis, but later in life, it promotes several age-related degenerative processes. For instance, AhR factor (i) controls the pluripotency of stem cells and the stemness of cancer stem cells, (ii) it enhances the differentiation of embryonal stem cells, especially AhR signaling modulates the differentiation of hematopoietic stem cells and progenitor cells, (iii) it also stimulates the differentiation of immunosuppressive Tregs, Bregs, and M2 macrophages, and finally, (iv) AhR signaling participates in the differentiation of many peripheral tissues. On the other hand, AhR signaling is involved in many processes promoting cellular senescence and pathological processes, e.g., osteoporosis, vascular dysfunction, and the age-related remodeling of the immune system. Moreover, it inhibits autophagy and aggravates extracellular matrix degeneration. AhR signaling also stimulates oxidative stress, promotes excessive sphingolipid synthesis, and disturbs energy metabolism by catabolizing NAD^+^ degradation. The antagonistic pleiotropy of AhR signaling is based on the complex and diverse connections with major signaling pathways in a context-dependent manner. The major regulatory steps include, (i) a specific ligand-dependent activation, (ii) modulation of both genetic and non-genetic responses, (iii) a competition and crosstalk with several transcription factors, such as ARNT, HIF-1α, E2F1, and NF-κB, and (iv) the epigenetic regulation of target genes with binding partners. Thus, not only mTOR signaling but also the AhR factor demonstrates antagonistic pleiotropy in the regulation of the aging process.

## Introduction

**Fig. 1:**
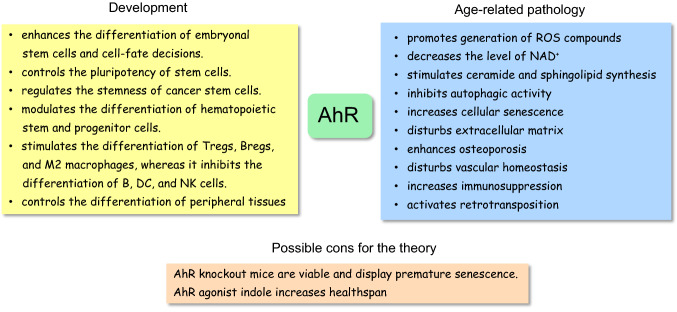
AhR signaling represents an example of antagonistic pleiotropy in the regulation of the developmental and aging processes. Major beneficial developmental and harmful age-related properties have been listed for comparison. There are some cons for the theory which have been discussed in the text

The aryl hydrocarbon receptor (AhR) is an evolutionarily conserved transcription factor which first appeared over 600 million years ago [1]. The AhR factor is a ligand-regulated transcription factor which is an ancient member of the basic-helix/loop/helix per Arnt-sim (bHLH/PAS) family [2]. Originally, the AhR factor was studied as an environmental sensor for many xenobiotics, such as 2,3,7,8-tetrachlorodibenzo-p-dioxin (TCDD) and polycyclic aromatic hydrocarbons (PAH). Currently, it is known that the AhR factor is not only involved in chemical defence, but it has also a crucial role in developmental biology and in the function of the immune system [3–5]. For instance, AhR signaling regulates the pluripotency of embryonic stem cells and affects their differentiation into diverse tissue types (Fig. 1). Moreover, Ahr signaling controls the differentiation of immune cells, especially enhancing the generation of immunosuppressive phenotypes (see below). On the other hand, with aging, AhR signaling increases cellular senescence and osteoporosis, inhibits autophagy, and disturbs vascular homeostasis (Fig. 1). Interestingly, the function of the AhR factor is a good example of antagonistic pleiotropy, i.e., a particular gene has crucial functions during development, but its activity evokes detrimental responses later in the life [6]. Here, I will shortly introduce the theory of antagonistic pleiotropy and then describe in detail many important functions of the AhR factor during embryogenesis and for comparison, examine its role as an enhancer of age-related degenerative processes.

## AhR-driven antagonistic pleiotropy in development and aging process

The antagonistic pleiotropy hypothesis is a well-known evolutionary theory explaining the aging process, originally proposed by George Williams in 1957 [6, 7]. The antagonistic pleiotropy theory postulates that a particular gene regulates several functional traits which support the developmental process, but which exert detrimental effects during the aging process. The theory predicts that natural selection favors the vigor of youth over the frailty of old age. Those genes revealing characteristics of antagonistic pleiotropy enhance reproduction and growth at a young age, but these same genes have deleterious effects later in life promoting the aging process. The theoretical basis underpinning the antagonistic pleiotropy and its functional explanations in the aging process have been reviewed elsewhere in detail [6, 8]. The disposable soma theory, adapted from the antagonistic pleiotropy theory, suggests that organisms have a limited amount of resources which are allocated to reproduction and growth at the cost of repair processes during the aging process [9]. Subsequent studies on genetics, e.g., on the long-lived mutants, have provided novel insights into the evolutionary mechanisms controlling the aging process [8, 10]. For instance, the *age-1* and *daf-2* mutants in *Caenorhabditis elegans* as well as the *chico* and *Inr* mutants in *Drosophila* increased lifespan, but correspondingly, they exhibited a strongly reduced reproduction or sterility in *C. elegans* and *Drosophila* [8]. These genes are driving the insulin/insulin-like growth factor (IGF) receptor signaling pathway. In mammals, this pathway activates the mammalian target of rapamycin (mTOR) pathway which is known to display antagonistic pleiotropy in the regulation of the aging process [11, 12]. The insulin/IGF-1/TOR axis undertakes several crucial functions during reproduction and the growth of the organism, whereas the same pathway has many detrimental effects during the aging process. For instance, the inhibition of autophagy with aging increases the accumulation of garbage into aged cells [12]. Interestingly, there is clear indication that AhR signaling also inhibits autophagy, but it seems that it is mediated through the E2F1 signaling rather than the insulin/IGF1/mTOR signaling pathway (see below).

### AhR stimulates developmental processes

Across the evolution from single-cell organisms to multicellular animals, AhR signaling has undertaken functions far beyond its role as an environmental sensor (Fig. 1). For instance, it is known that AhR signaling is important in many developmental processes and in the immune defence of the organism [5, 13, 14]. During embryogenesis, the expression of the *AhR* gene displays significant stage-specific alterations [14–16]. The *AhR* gene was strongly expressed in mouse fertilized eggs and up to the four-cell morula stage, but afterwards the expression level disappeared during the cleavage phase until it became evident again in the early blastocyst. After implantation, the expression of the *AhR* gene increased although it displayed cell type- and tissue-specific expression levels. Interestingly, a global decline in the expression of mouse *AhR* gene during the morula and early blastula phase coincided with a decrease in the DNA methylation state during increased proliferation [14]. Studies conducted in the AhR-knockout mice have revealed significant developmental perturbations in some tissues, especially in liver, spleen, and cardiac muscle, as well as some crucial impairments in their immune system [17–19]. Fernandez-Salguero et al. [20] reported that the AhR-null (*AhR*^*−/−*^) mice were viable and fertile, but had a 45% mortality and displayed clearly a sick phenotype by 13 months of age. Intriguingly, the constitutively active *AhR* gene (CA-AhR) expressed in transgenic mice also impaired the developmental processes of liver and kidney [21], disturbed neurogenesis [22], and increased the risk of cancers, e.g., in stomach [23]. It is clear that the *AhR* gene performs many homeostatic functions in the developmental processes that explain why its expression is stringently regulated.

Currently, there is convincing evidence that AhR factor has an important role in the regulation of pluripotency of embryonal stem (ES) cells since it promotes their differentiation into diverse cell lineages (Fig. 1). Ko et al. [24] demonstrated that several pluripotency factors, i.e., OCT4, NANOG, and SOX2, were able to inhibit the expression of *AhR* gene in mouse ES cells by binding to the silencer domain of the *AhR* gene. Several other investigators have also revealed that the *AhR* gene needs to be repressed so that ES cells can maintain their mitotic progression and pluripotency [16, 25]. For instance, OCT4 and NANOG factors displayed an overexpression in the mouse *AhR*−/− embryos inhibiting their differentiation process [16]. On the other hand, an increased activation of AhR factor inhibited the expression of OCT4 and NANOG and consequently promoted the differentiation of mouse ES cells [5, 14, 16]. Recently, Gonzalez-Rico et al. [26] demonstrated that AhR factor was able to bind to two Alu elements flanking the human *NANOG* gene thus assembling a chromatin loop which inhibited the expression of the *NANOG* gene. This AhR-driven retrotransposon-mediated chromatin modification inhibited the expression of human NANOG factor and subsequently induced the differentiation of human NTERA-2 cells. Moreover, Cheng et al. [27] demonstrated that the activation of AhR factor induced the binding of AhR factor to the promoter of human *OCT4* gene, inhibited its expression, and consequently induced the differentiation of several cancer stem-like cells, thus reducing their tumorigenic potential. However, several studies have indicated that the expression of AhR maintained and enhanced the stemness of cancer stem cells. For instance, Stanford et al. [28] revealed that an increased expression of AhR factor augmented the development of human cells with cancer stem-like properties which enhanced tumorigenesis by increasing their migration and invasion. Yan et al. [29] also reported that the activation of AhR signaling in the radioresistant human epithelial cancer cells induced the expression of genes associated with the stem-like phenotype, e.g., *ABCG2*, *c-MYC*, and *CXCR4* genes. They also revealed that nuclear IKKα protein was involved in the AhR-mediated regulation of stemness in human epithelial cancer cells.

AhR factor has a crucial role in the maintenance of hematopoietic stem cells (HSC) and progenitor cells and subsequently it is involved in their differentiation to a diverse set of immune cells in a cell-specific manner [4, 30, 31]. There is clear evidence that the activation of AhR factor is a negative regulator of hematopoiesis inhibiting excessive proliferation and thus it maintains homeostasis in the immune system [30, 32, 33]. Vaughan et al. [33] demonstrated that the inhibition of AhR signaling in mice through its knockout or the antagonist treatments induced a myeloid-biased increase in HSCs and progenitor cells. There also appeared an increase in the frequency of progenitors committed to pregranulocyte/premonocyte lineages. The role of AhR signaling has been elucidated in the differentiation of the progenitor cells into effector and regulatory immune cells. For instance, Li et al. [34] revealed that an increased AhR signaling impaired the human B cell lymphopoiesis from hematopoietic stem cells to early-B and pro-B cells. AhR signaling also disturbed the differentiation of human natural killer (NK) cells [35] and mouse dendritic cells (DC) [36]. Interestingly, Tousif et al. [37] demonstrated that AhR signaling promoted the differentiation of B cells into the regulatory B (Breg) cells in a mouse model of lung cancer. In addition, there is convincing evidence that an activation of AhR signaling induced the differentiation of mouse T cells into regulatory T (Treg) cells [38, 39] (Fig. 1). It seems that an increase in AhR signaling disturbs the function of effector immune cells, whereas it augments immunosuppressive phenotypes.

The developmental regulation of AhR signaling is not limited to the embryonal differentiation or the hematopoiesis, but it also affects the terminal differentiation and the growth of several tissues, e.g., neurogenesis, cardiovascular development, and osteogenesis [40–42]. In this respect, it seems that AhR signaling has both beneficial and detrimental effects in a context-dependent manner during development. It is also evident that different models, i.e., the knockout/antagonist or the overexpression/agonist treatments, generate non-physiological responses in long-term experiments. Neurogenesis has been frequently studied with the aim of clarifying the role of AhR signaling during development of the brain. Latchney et al. [40] reported that both the deletion and the activation of AhR signaling disturbed neurogenesis and impaired memory and cognition in growing mice. The 3-month-old AhR-deficient mice experienced an abnormal neuronal differentiation and a reduced cell survival. The AhR-null mice also revealed a reduced myelination of neurons [43]. On the other hand, Kimura et al. [22] reported that the long-term activation of AhR (CA-AhR) impaired the dendritic growth and the positioning of cortical mouse pyramidal neurons. Recently, Wei et al. [44] demonstrated that certain tryptophan-metabolizing microbes in the gut were able to enhance mouse hippocampal neurogenesis by promoting synaptic maturation and activity via AhR signaling. These studies indicate that AhR signaling is crucial for neurogenesis although the overexpression of AhR factor disturbs the programmed development of brain.

There is clear evidence that AhR signaling affects the development of peripheral tissues. For instance, studies on cardiovascular development have revealed that there is an abnormal morphogenesis induced by either AhR ablation or agonist treatments [41]. Disturbances involved alterations, e.g., in tissue structures, cellular metabolism, and cardiac functions leading to defects similar to those encountered in congenital heart disease (CHD). Pulignani et al. [45] reported that the presence of the genetic variant Arg554Lys in the AhR protein was associated with an increased risk of CHD. It is known that an AhR-deficiency can induce cardiac hypertrophy and increase arterial blood pressure in mice [46]. The activation of AhR signaling also regulates the development of bones and kidneys. Osteogenesis of the bones involves a balance between the activities of osteoblasts and osteoclasts during the developmental phase and this continues to ensure the maintenance of bone homeostasis later in the life. There is abundant evidence indicating that AhR signaling suppresses osteoblastogenesis, whereas it increases osteoclastogenesis during the development and remodeling of the bone [42, 47]. Izawa et al. [42] demonstrated that the overexpression of AhR signaling in mice enhanced osteoclastogenesis which promoted osteoporosis with aging. On the other hand, treatments with antagonists of AhR signaling, e.g., isopsoralen, stimulated the differentiation of osteoblasts and subsequently increased bone mineralization [48]. Falahatpisheh et al. [49] demonstrated that the expression of AhR was crucial for the development of mouse kidneys. They reported that AhR signaling regulated signaling via the Wilms’ tumor suppressor 1 (WT1) during nephrogenesis, especially affecting the development of kidney glomeruli. In conclusion, it seems that a constant activity of AhR signaling maintains developmental homeostasis in the tissues, and thus either a decline or an excessive activation of AhR signaling impairs embryogenesis.

### AhR promotes the age-related degenerative processes

Several aging studies have revealed that AhR signaling is associated with many age-related degenerative processes [50, 51] (Fig. 1). It appears that some of the properties driven by AhR are crucial in the early growth of embryos, but subsequently become harmful during the aging process, e.g., the repression of autophagy enhances the growth of tissues during embryogenesis, but it disturbs cellular homeostasis with aging [52]. Moreover, apoptosis and cellular senescence are important tissue remodeling mechanisms during development, but the accumulation of senescent cells with aging enhances the aging process [53, 54]. The expression of matrix-degrading metalloproteinases (MMP), a function controlled by AhR signaling, has an important role in the development of tissues during embryogenesis, but with aging, an increased expression of MMPs has many harmful effects on tissue integrity [55]. The increased inflammatory microenvironment with aging enhances the activity of AhR signaling which might promote the age-related remodeling of the immune system (see below). It seems that certain properties of AhR factor are driving the growth of organism, but they have detrimental effects with aging.

It has been difficult to obtain direct evidence on the role of AhR activity in the aging process because both the depletion and overexpression of mammalian AhR factor disturb the physiological functions of AhR signaling causing pathological consequences and leading to premature aging. One reason might be the fact that the expression of the *AhR* gene is stringently regulated in a context-dependent manner. In addition, it is not only the expression level of AhR factor since a number of endogenous and exogenous ligands, either agonists or antagonists, regulate the context-dependent activity of AhR factor. However, the genetic models of *Caenorhabditis elegans* have been exploited in the lifespan studies on the mutants of the *ahr-1* gene. For instance, Eckers et al. [56] demonstrated that the *ahr-1* (*ju 145*) mutants displayed an increased mean lifespan as well as they revealed an improved motility and heat resistance. However, Sonowal et al. [57] reported that certain indoles, AhR agonists, from commensal bacteria extended the healthspan of many organisms, e.g., *C. elegans*, *Drosophila*, and mice, although indoles did not affect maximum lifespan (Fig. 1). On the other hand, the extrinsic activation of AhR factor in mouse skin promoted the aging process [50]. It is known that chronic UV radiation (UVR) can induce the AhR-mediated photoaging in the skin [58]. Next, I will examine the properties of AhR signaling which are able to generate the essential hallmarks of the aging process.

#### AhR signaling generates diverse cellular stresses

**Fig. 2:**
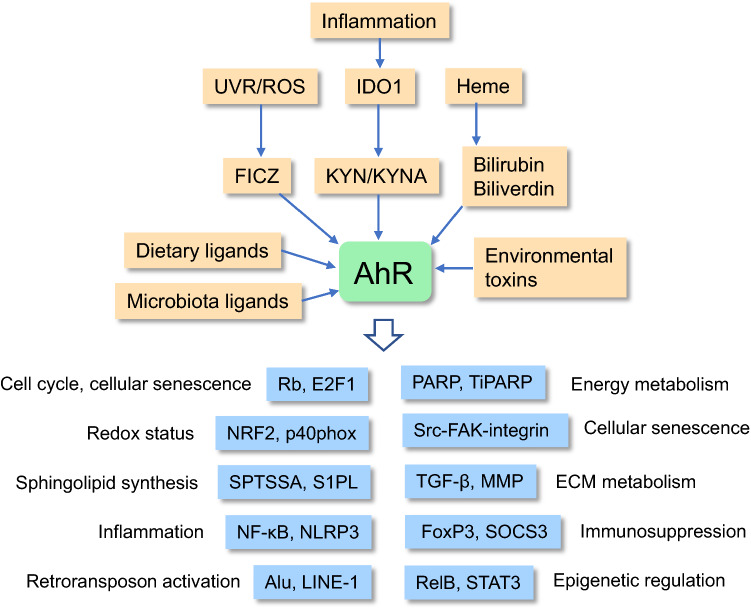
Age-related properties induced by the ligand-activated AhR signaling. A large variety of endogenous and exogenous ligands activate AhR signaling which promotes the aging process in a context-dependent manner. Certain dietary, environmental, and microbiota ligands can be antagonists for AhR activation (see text). *E2F1* E2F transcription factor 1, *FAK* focal adhesion kinase, *FICZ* 6-formylindolo[3,2-b]carbazole, *FoxP3* forkhead box P3, *IDO1* indoleamine 2,3-dioxygenase, *KYNA* kynurenic acid, *KYN* kynurenine, *MMP* matrix metalloproteinase, *NF-κB* nuclear factor-κB, *NLRP3* NOD- LRR- and pyrin domain-containing protein 3, *NRF2* nuclear factor-erythroid factor 2-related factor 2, p40phox p40 component of NADPH oxidase, *PARP* poly(ADP-ribose) polymerase, *Rb* retinoblastoma, *RelB* RELB proto-oncogene, *ROS* reactive oxygen species, *SOCS3* suppressor of cytokine signaling 3, *SPTSSA* serine palmitoyltransferase small subunit A, *Src* SRC proto-oncogene, *STAT3* signal transducer and activator of transcription 3, *TiPARP* TCDD-inducible poly(ADP-ribose) polymerase, *TGF-β* transforming growth factor-β, *UVR* ultraviolet radiation

Increased AhR signaling can induce many cellular stresses, such as oxidative stress, sphingolipid accumulation, and the depletion NAD^+^, all of which disturb cellular functions and enhance the aging process (Fig. 1). Interestingly, it is known that oxidative stress is a potent enhancer of the aging process, but it is an important mechanism during the embryonic development phase [59]. Particularly, redox regulation has a crucial role in the self-renewal and lineage commitment of stem cells [60, 61]. ROS compounds can control the activity of many key transcription factors associated with both the developmental and the aging processes, e.g., NF-κB, HIF-1α, and NRF2. ROS compounds regulate not only the development of stem cells, but also organismal aging in a concentration-dependent manner [62]. Low concentrations induce adaptive responses, whereas higher concentrations trigger pathological changes. There is convincing evidence that the activation of AhR signaling generates ROS compounds [63]. Certain target genes of AhR transcription factor are associated with the generation of ROS/oxidative stress, e.g., cytochrome P450 family 1 subfamily A member 1 (CYP1A1) and the p40phox component of NADPH oxidase [63, 64] (Fig. 2). CYP1A1, a member of cytochrome P450 family, is located in mitochondria and is involved in the production of superoxide in mitochondria [63]. The p40phox component has an important role in the ROS production by NADPH oxidase [64]. AhR signaling promotes many age-related processes via the generation of ROS compounds, e.g., inflammatory responses, cellular senescence, and remodeling of ECM (see below). As a pleiotropic factor, AhR can also activate the antioxidant pathways which have an important role in the maintenance of the cellular redox status [65]. In fact, the AhR-NRF2 signaling is the best characterized of the antioxidant pathways driven by AhR signaling (Fig. 2). It is known that ROS compounds can control both the developmental and the aging processes through an epigenetic regulation of the chromatin landscape [66].

Sphingolipids are important membrane components, but they also have many signaling functions, e.g., they are involved in the regulation of cell proliferation, adhesion, autophagy, apoptosis, and many immune functions [67]. Sphingolipids have a crucial role in the developmental processes including brain development and stem cell differentiation [68, 69]. On the other hand, sphingolipids, especially ceramide, regulates cellular senescence and the aging process [70, 71]. Majumder et al. [72] utilized a genome-wide CRISPR/Cas9 screening to demonstrated that AhR factor increased the levels of several sphingolipids in mouse liver and lung. AhR factor induced the transcription of mouse *serine palmitoyltransferase small subunit A* (SPTSSA) gene. The SPT complex is the first and rate-limiting step in the synthesis of sphingolipids (Fig. 2). Moreover, Wang et al. [73] reported that AhR factor inhibited the expression of human sphingosine-1-phosphate lyase (S1PL) enzyme which controls the degradation of S1P to phosphoethanolamine. This means that the activation of AhR signaling is able not only to enhance the synthesis of sphingolipids, but also to inhibit their degradation. Ceramide and S1P are the most important signaling components of sphingolipids and they have many opposing functions, called the sphingolipid rheostat. For instance, ceramides induce cell-cycle arrest, cellular senescence, apoptosis, and neurodegeneration, whereas S1P has many beneficial properties, e.g., it controls lymphocyte differentiation and trafficking as well as it promotes neuroprotection [74, 75]. Different approaches have revealed that the level of ceramides increases with aging, thus probably promoting senescence and the aging process [70, 71]. Interestingly, Huang et al. [76] demonstrated that the inhibition of SPT reduced sphingolipid synthesis and significantly extended the lifespan of yeast. Given that AhR signaling stimulated the expression of SPT, this might accelerate the aging process of mammals. Currently, it is known that the AhR-induced activation of SPT is associated with the appearance of many age-related diseases, e.g., hepatic lipid accumulation in mice [77].

The maintenance of energy homeostasis is crucial for both developmental processes and healthspan extension. NAD^+^ is a coenzyme which not only controls energy status, but it also supplies the NAD^+^-consuming enzymes, e.g., poly(ADP-ribose)polymerases (PARP) and sirtuins (SIRT1-7) [78]. Interestingly, the activation of AhR factor can disturb energy balance by reducing the cellular level of NAD^+^. Ma [79] was the first investigator who demonstrated that TCDD induced the AhR-mediated expression of a novel PARP enzyme, called TiPARP (ARTD14/PARP7). Subsequently, MacPherson et al. [80] revealed that human TiPARP enzyme was a mono-ADP-ribosyltransferase which also inhibited the transactivation of the *AhR* gene. This shows a negative feedback loop in AhR signaling. The TiPARP enzyme has an important role in the developmental process since the TiPARP knockout mice experienced disturbances in the development of GABAergic neurons and the loss of TiPARP induced an abnormal layering of mouse cerebral cortex [81]. There is convincing evidence that the activation of AhR factor reduced the level of NAD^+^ under diverse experimental conditions [82]. It seems that AhR signaling decreases the level of NAD^+^ attributed to the activation of TiPARP, whereas concurrently it increases the mono-ADP-ribosylation of many proteins, thus affecting the maintenance of homeostasis. There is robust evidence that the cellular level of NAD^+^ declines in senescent cells and during aging in many tissues [83, 84]. Currently, the role of AhR signaling in these age-related alterations is not completely clear.

#### AhR signaling promotes cellular senescence and controls apoptosis

The accumulation of senescent cells into tissues is a major hallmark of the aging process [85]. Senescent cells undergo an irreversible arrest of cell-cycle which has been attributed to an activation of several tumor suppressors, such as p53, p16Ink4a, p21Cip1, p27Kip1, and retinoblastoma (Rb) protein [85, 86]. There is abundant evidence that the activation of AhR signaling by several agonists arrests the proliferation of different cell types and consequently triggers a senescent phenotype [87–89]. Interestingly, AhR signaling also inhibits the proliferation of stem cells, e.g., embryonal stem cells [25], hematopoietic stem cells [90], and bone marrow mesenchymal stem cells [89] (Fig. 2). It seems that there are several mechanisms through which AhR factor can repress the cell-cycle progression. For instance, AhR factor can increase the expression of p21Cip1 and p27Kip1 proteins and thus halt cell proliferation. Jackson et al. [91] demonstrated that the activation of AhR by TCDD induced the expression of p21Cip1 protein and consequently inhibited the regeneration of mouse liver. The p21Cip1 inhibited the G1-phase cyclin-dependent kinase 2 (CDK2) activity and induced a G0/G1 cell-cycle arrest. Accordingly, Kolluri et al. [92] reported that the activation of AhR factor induced the expression of p27Kip1 protein and suppressed the proliferation of developing mouse thymus and rat hepatoma cells. There are several studies indicating that the AhR protein can bind to the Rb protein and consequently repress the function of E2F transcription factors, thus inhibiting DNA synthesis and cell-cycle progression [93, 94] (Fig. 2). Marlowe et al. [94] revealed that the binding of AhR protein to E2F transcription factors displaced the p300 protein from the complex and subsequently inhibited the transcription of many E2F-regulated genes which control the S phase progression. It is known that the E2F1 transcription factor inhibits the Forkhead box O transcription factors (FoxO) and thus controls cellular senescence and organismal aging [95]. Moreover, the aging process is associated with a progressive decline in the numbers of stem cells [96]. Currently, the role of AhR signaling in the exhaustion of age-related stem cells needs to be clarified.

Programmed cell death, i.e., apoptosis, is a crucial architect of mammalian development [54]. Although AhR signaling has a fundamental role in embryogenesis, there are only a few reports on its effects on apoptosis during developmental processes. For instance, the Bax-dependent apoptosis driven by AhR signaling controlled the development of murine fetal ovarian germ cells [97]. However, there is a substantial literature examining the role of AhR signaling in apoptosis, both in pro-apoptotic and anti-apoptotic responses, later in the life. It seems that the activation of AhR signaling by environmental toxins, e.g., TCDD and polycyclic aromatic hydrocarbons (PAH), induces an apoptotic cell death in diverse cell types [98, 99]. There appears to be several mechanisms in the toxin-induced apoptosis although the ROS-induced mitochondrial dysfunction seems to be the major process. However, it is not known how AhR signaling is driving the endogenously-induced apoptosis or resistance to apoptosis. For instance, it has been demonstrated that C2-ceramides can trigger apoptosis in murine hepatomas [100]. On the other hand, there is robust evidence that AhR signaling can prevent apoptotic cell death in many tissues. As a proof of principle, Elizondo et al. [101] reported that embryonic fibroblasts obtained from the AhR-knockout mice were predisposed to apoptosis in cell culture. Moreover, when AhR signaling was activated by tumor promoters, this proved to be a potent inhibitor of apoptosis and an effective inducer of tumorigenesis [102]. For instance, Bekki et al. [103] demonstrated that AhR signaling activated by kynurenine (KYN) exposure enhanced apoptosis resistance in human breast cancer cells. Interestingly, there is clear evidence indicating that the resistance to apoptosis increases in both cellular senescence and the aging process [104, 105]. Marlowe et al. [106] demonstrated that the AhR protein formed a complex with the E2F1 factor and thus inhibited the E2F1-induced apoptosis in mouse hepatoma cells and human osteosarcoma cells. Currently, it needs to be clarified whether the resistance to the apoptosis associated with aging and cellular senescence is related to AhR signaling. It is clear that any decline in the extent of apoptosis with aging would prevent the elimination of unfit cells and thus disturb tissue homeostasis.

#### AhR signaling inhibits autophagic degradation

Autophagy is a catabolic process which involves the degradation of intracellular proteins and organelles via the lysosomal pathway. Mammalian target of rapamycin (mTOR), a major regulator of protein synthesis and cellular growth, is a potent inhibitor of autophagy. Schmeisser and Parker [12] have reviewed the potential role of mTOR-dependent autophagy as a model of antagonistic pleiotropy. A low level of autophagy enhances the growth of tissues during development, but with aging, it has detrimental effects on tissue homeostasis. There is robust evidence that autophagic degradation declines with aging and that there are disturbances in the function of the autophagy-lysosome pathway [52, 107]. Interestingly, there are observations indicating that AhR signaling is able to suppress autophagic activity. Kondrikov et al. [89] demonstrated that KYN, an endogenous agonist for AhR, inhibited autophagy via the activation of AhR signaling in the bone marrow mesenchymal stem cells (BMSC) obtained from aged mice. The physiological levels of KYN disrupted autophagic flux and inhibited macroautophagy induced by serum-starvation in mouse BMSCs. Kim et al. [108] reported that the activation of AhR signaling with TCDD and proinflammatory cytokines in human keratinocytes decreased the expression of several autophagy-related factors including Beclin 1, ATG5, and LC3 proteins. The production of LC3-positive autophagosomes was also down-regulated in activated keratinocytes. Surprisingly, the inhibition of autophagy with chloroquine increased the expression of AhR in the cytokine-stimulated keratinocytes. Yang and Chan [109] also observed that the inhibition of autophagy by chemical agents increased the level of the AhR protein in human HeLa cells. They revealed that the AhR protein was degraded through the p62/LC3-mediated selective autophagy in diverse human cell lines. Thus, the inhibition of autophagy, e.g., by mTOR signaling, enhances the AhR-related responses. Currently, the mechanism of the AhR-induced repression of autophagy still needs to be clarified. Polager et al. [110] demonstrated that the E2F1 factor stimulated autophagy by increasing the expression of several autophagy genes, e.g., *ATG1*, *ATG5*, *LC3, and DRAM* genes, in human osteosarcoma cells. This means that the E2F1 factor does not only arrest cell cycle and inhibit apoptosis, but it also stimulates autophagy in order to maintain homeostasis. As discussed earlier, AhR is a potent inhibitor of E2F1 signaling [93, 94, 106] and thus it could promote the age-related inhibition of autophagy. One might speculate that the AhR factor, as an effective growth regulator during development, can inhibit certain catabolic activities with aging although they are crucial for the maintenance of cellular integrity, i.e., autophagy and apoptosis. The inhibition of autophagy by AhR signaling is a good example of how antagonistic pleiotropy can drive the aging process.

#### AhR signaling aggravates extracellular matrix degeneration

The extracellular matrix (ECM) has a crucial role in both the developmental morphogenesis of tissues and in the maintenance of homeostasis during the aging process. Tissue development is dependent on a constant remodeling of ECM structures involving a dynamic balance between both synthetic and catabolic processes [111]. ECM provides the microenvironment for both cellular differentiation and tissue morphogenesis during embryogenesis. This developmental process is regulated by a diverse network of signaling mechanisms and the enzymes controlling the homeostasis of ECM. On the other hand, it is known that the aging process is associated with many degenerative activities in the ECM of many tissues, e.g., in the skin and cardiac muscle [112, 113]. For example, there is an increased expression and activity of many matrix metalloproteinases (MMP), an accumulation of collagen with an increased level of cross-linking, and an enhanced fragmentation of ECM components; these are all common hallmarks of age-related degeneration of ECM integrity. Moreover, the degeneration of ECM modifies immune responses thus exposing tissues to inflammation and many diseases [114]. Interestingly, AhR signaling has an important role in the ECM remodeling and the maintenance of its homeostasis [115, 116]. It is known that AhR signaling stimulates the expression and activity of different MMPs, e.g., MMP1 and MMP9, and thus enhances the degradation of ECM structures in a context dependent manner (Fig. 2). There is robust evidence indicating that the expression of many MMPs increases with aging in different tissues [113]. AhR signaling co-operates with several signaling pathways, such as TGF-β, NF-κB, and integrin pathways, in the regulation of cell adhesion and ECM metabolism [116, 117]. The TGF-β cytokine is a pleiotropic regulator of ECM remodeling, i.e., its signaling is stringently controlled in a cell-specific manner [117, 118]. However, AhR and TGF-β factors display mutually repressive signaling mechanisms in many conditions [117]. For instance, AhR signaling regulates the expression of the latency-associated protein 1 (LTBP-1) which inhibits the activation of latent TGF-β in the ECM. This regulation is under the epigenetic control and is thus a context-dependent process. It has been proposed that the AhR/LTBP-1/TGF-β axis has a crucial role in many pathological conditions [117]. Tomkiewicz et al. [119] demonstrated that the activation of AhR factor stimulated the Src kinase which subsequently activated focal adhesion kinase (FAK) and enhanced cell migration in human HepG2 cells. Moreover, TCDD exposure increased the expression of several integrin proteins, e.g., ITGβ1, ITGβ3, and ITGα1, in human HepG2 cells. Interestingly, Rapisarda et al. [120] revealed that an increased expression of ITGβ3 induced the senescence of several human cell types by activating TGF-β signaling. In this respect, the activation of AhR signaling enhances cell migration during the developmental phase as well as cell senescence and ECM degradation with aging.

#### AhR signaling provokes osteoporosis and vascular dysfunction

AhR signaling has an important role in the control of homeostasis in bones and vascular tissues. The balance between the activities of osteoblasts and osteoclasts regulates the mineralization and resorption of the bone [121]. There is abundant evidence that the activation of AhR signaling arrests the proliferation of human osteoblasts [122], whereas it stimulates the formation of osteoclasts thus increasing bone resorption and enhancing osteoporosis with aging [42, 47, 123]. Eisa et al. [47] reported that KYN, an agonist of AhR, induced the generation of osteoclasts from mouse macrophages via the activation of the receptor of nuclear factor κB ligand (RANKL) signaling. KYN treatment also enhanced the resorption of mouse bone. However, recent studies have revealed that the AhR-driven osteoclastogenesis in mouse macrophages is dependent on both the concentration of indoxyl sulfate, an endogenous AhR agonist, as well as the duration of treatment [124]. A short-term and low-dose exposure stimulated mouse osteoclast differentiation, whereas a long-term and high-dose treatment inhibited the differentiation of mouse macrophages into osteoclasts. Refaey et al. [125] reported that feeding of adult mice with KYN increased the serum level of RANKL, a marker for osteoclastic activity, as well as it induced osteoporotic changes in the bone microarchitecture. KYN supplementation also augmented the adiposity of bone marrow. It is known that the activity of indoleamine 2,3-dioxygenase 1 (IDO1) and consequently the serum levels of KYN significantly increase with aging [126, 127]. Accordingly, Chung et al. [128] reported that the aging process notably upregulated the markers of osteoclastogenesis in human bone marrow cells, such as the expression levels of *RANK* and *c-fms* genes. Currently, the mechanisms behind the pleiotropic responses of AhR signaling in the maintenance of bone homeostasis remain to be elucidated.

AhR signaling regulates not only vascular development, but also sprouting angiogenesis which can have either beneficial or detrimental effects in diverse diseases. It is known that different AhR agonists inhibit the proliferation, migration, and tube formation of human umbilical vein/artery endothelial cells (HUVEC/HUAEC) [129]. Ichihara et al. [130] demonstrated that a hindlimb ischemia induced a markedly increased angiogenesis in the AhR-knockout mice in comparison with that of their normal counterparts. The enhanced angiogenesis was associated with an increased expression of hypoxia-inducible factor-1α (HIF-1α) which stimulated the expression of vascular endothelial growth factor (VEGF) subsequently enhancing angiogenesis. Given that AhR and HIF-1α factors share the same nuclear transporter ARNT protein [131], it seems that a reduced level of AhR factors could well increase the nuclear transport of HIF-1α (see below). It is known that exposure to indoxyl sulfate was able to promote rat endothelial senescence [132] and enhance the disruption of rat blood–brain barrier [133] via an activation AhR signaling. Indoxyl sulfate, a uremic toxin produced from L-tryptophan, has been claimed to be associated with some serious vascular diseases, e.g., chronic kidney disease and cardiovascular disease [134]. Eckers et al. [56] demonstrated that the overexpression of AhR factor or its stimulation with agonists impaired the activation of endothelial nitric oxide synthase (eNOS) and the production of NO in human endothelial cells. eNOS has a crucial role in the maintenance of vascular homeostasis and it is evident that its reduced activation could lead to the development of vascular diseases [135].

#### AhR signaling induces the remodeling of immune system

The aging process is associated with a significant remodeling of both arms of the immune system, i.e., adaptive and innate immunity [136–138]. Common hallmarks of immunoaging include (i) an involution of the thymus, (ii) a chronic low-grade inflammation called inflammaging, (iii) increased immunosuppressive activity attempting to counteract inflammaging, and (iv) immunosenescence of effector immune cells reducing the functional activity of the immune system with aging. Interestingly, AhR signaling seems to be able to regulate these processes in a context-dependent manner. It is known that the activation of AhR signaling can promote an atrophy/involution of mouse thymus which disturbs the maturation of lymphocytes with aging thus enhancing immunosenescence [82, 139]. Laiosa et al. [139] revealed that the activation of AhR signaling within intrathymic lymphocyte progenitor cells arrested their proliferation and consequently induced the atrophy of mouse thymus. Diani-Moore et al. [82] reported that the stimulation of AhR signaling by TCDD activated poly (ADP-ribose) polymerase (TiPARP) which caused the loss of NAD^+^ and subsequently elicited thymic atrophy in mice. The exposure to a PARP inhibitor increased the level of NAD^+^ and prevented the atrophy of thymus. Age-related involution of thymus is an important contributor to inflammaging and immunosenescence [140].

There is clear evidence that AhR signaling can induce both pro-inflammatory and anti-inflammatory responses through different signaling pathways [4, 141, 142]. Commonly, many environmental pollutants, e.g., polycyclic aromatic hydrocarbons (PAH), are known to stimulate oxidative stress and induce inflammatory responses [143, 144]. The AhR-induced ROS production probably is the major mechanism underpinning the generation of inflammation since ROS are well-known inducers of inflammatory responses [145]. Vogel et al. [144] demonstrated that inflammatory stimuli also induced the expression of AhR factor in human dendritic cells. They revealed that the promoter of the human *AhR* gene contained three putative NF-κB binding sites; of these, one motif mediated the RelA/p50-induced transcription of the *AhR* gene which explains why an increased NF-κB signaling is associated with the stimulation of AhR signaling. Subsequently, AhR signaling is able to control the function of a large set of lymphoid cells generating either pro- or anti-inflammatory responses [4, 146]. Moreover, bilirubin and biliverdin, the catabolic metabolites of heme protein, are also agonists of the AhR factor [147] (Fig. 2). It is known that diverse stresses can increase the levels of these compounds which possess anti-inflammatory activity by inhibiting the activation of NF-κB and inflammasomes [148].

There is convincing evidence that AhR signaling is driving an anti-inflammatory regulation rather than pro-inflammatory responses. Huai et al. [149] demonstrated that the AhR factor suppressed the function of inflammasomes by inhibiting the transcription of the *NLRP3* gene in mice. They revealed that AhR factor was able to bind to the xenobiotic response element (XRE) in the promoter of the *NLRP3* gene and inhibited its transcription (Fig. 2). Inflammasomes are responsible for the activation of a diverse set of inflammatory responses driven by the innate immune system. AhR factor can also suppress inflammatory responses by inducing the expression of SOCS3 protein, a suppressor of cytokine signaling [150]. Wada et al. [150] revealed that the mouse and human promoter sequences of the *SOCS3* gene contained a transcriptionally active XRE site through which AhR factor inhibited the transactivation of the *SOCS3* gene in mouse liver and human HepG2 cells. They also reported that the activation of AhR signaling suppressed hepatic steatosis occurring when mice were fed a high fat diet. Moreover, the activation of AhR signaling stimulates the differentiation of immunosuppressive phenotypes of many immune cells, i.e., (i) induction of myeloid-derived suppressor cells (MDSC) [151], (ii) Tregs [38], (iii) Bregs [37], (iv) tolerogenic dendritic cells (tolDC) [152], and (v) M2 macrophages (Mreg) [153]. For instance, AhR signaling stimulated the expression of Forkhead box 3 (FoxP3) protein, a master regulator of Tregs [38] (Fig. 2). AhR factor can also induce immunosuppressive responses via the non-genomic Src-STAT3 pathway. Zhu et al. [154] reported that AhR factor promoted the expression of IL-10 through the Src-STAT3 pathway in mouse inflammatory macrophages.

It is known that a chronic state of inflammation, such as inflammaging, triggers a compensatory immunosuppression which prevents excessive inflammatory responses [155]. There is clear evidence that the aging process induces the activation of immunosuppressive network which most probably counteracts inflammaging [138]. Interestingly, it seems that AhR signaling has a key role in the induction of inflammation-induced immunosuppression. It is recognized that NF-κB signaling, a major inducer of inflammatory responses, is also able to transactivate the expression of AhR factor [144]. Moreover, it is known that inflammation stimulates the expression of the IDO1 enzyme which activates the KYN pathway thus producing KYN and kynurenic acid (KYNA), potent agonists for AhR [39, 127] (Fig. 2). Consequently, the activation of AhR signaling can promote the expression of IDO1 [156], i.e., a positive feedback loop between IDO1 and AhR signaling. Given that AhR signaling stimulates anti-inflammatory/immunosuppressive responses (see above), this provides a counteracting regulation commonly observed not only in inflammaging [157], but also in age-related inflammatory diseases. An increased immunosuppressive activity with aging inhibits the functional activity of the immune system evoking a state called immunosenescence [158]. Immunosenescence in conjunction with immunosuppression increases a risk for cancers, enhances sensitivity to infections, and exposes elderly people to many age-related diseases [159]. For instance, it is well-known phenomenon that vaccination efficiency and the efficacy of immunotherapies decline as the individual enters old age. These observations are indications that there is a remodeling of the immune system with aging. Immunosuppressive mechanisms, e.g., the secretion of anti-inflammatory cytokines and ROS/RNS compounds as well as the depletion of certain amino acids via catabolism also promote degenerative bystander effects in inflamed host tissues [160]. For instance, TGF-β signaling can induce fibrosis, cellular senescence, osteoporosis, muscle atrophy, and alterations in ECM [161]. It seems that acute inflammatory responses stimulate the expression of AhR factor which consequently enhances immunosuppression and ultimately promotes immunosenescence and immunoaging of the immune system.

#### AhR signaling is increased in age-related inflammatory diseases

Currently, there are many review articles describing the role of AhR signaling in inflammatory diseases, e.g., atherosclerosis, neurodegenerative diseases, rheumatoid arthritis, and chronic infections [162, 163]. Given that AhR signaling has a crucial role in cardiovascular physiology, disturbances in its function have been linked with several cardiovascular diseases, e.g., atherosclerosis and ischemic heart disease [164]. For instance, AhR signaling can enhance inflammatory responses by promoting oxidative stress and cellular senescence in the intimal layer of the blood vessel wall. Moreover, Vogel et al. [165] demonstrated that TCDD stimulated the differentiation of human U937 macrophages into atherogenic foam cells. The foam cells secreted inflammatory mediators and MMPs thus disturbing ECM structures and inducing chronic inflammation in blood vessel wall. However, Kim et al. [166] reported that AhR signaling inhibited the transition of smooth muscle cells to chondromyocytes in human atherosclerotic lesions. This implies that AhR signaling has both beneficial and harmful effects in atherosclerotic tissue. There seem to exist tissue-specific differences in the AhR-induced responses to ischemic insults. Seong et al. [167] revealed that the activation of AhR signaling with an endogenous agonist, 2-[1′*H*-indole-3-carbonyl]-thiazole-4 carboxylic acid methyl ester (ITE), improved the function of mouse cardiac muscle after myocardial infarction. In fact, ITE treatment increased the number of immunosuppressive FoxP3-positive Tregs and shifted the pro-inflammatory M1 macrophages towards the anti-inflammatory M2 phenotype. On the other hand, Cuartero et al. [168] demonstrated that the activation of KYN/AhR pathway enhanced the extent of acute brain damage after middle cerebral artery occlusion (MCAO) in mice. An ischemic insult increased the nuclear translocation of AhR factor to neurons in peri-infarct and core regions. They also reported that the KYN-induced increase in infarct volume was AhR-dependent since it did not appear in the AhR antagonist-treated or in the AhR-knockout mice. Chen et al. [169] reported that AhR immunoreactivity mainly increased in activated microglia and astrocytes after MCAO in mice. They also observed that the activation of AhR after an ischemic stroke increased astrogliosis and suppressed neurogenesis in adult mice. These studies indicate that the responses of AhR signaling are context-dependent in inflammatory states.

Ramos-Garcia et al. [170] demonstrated that the expression of AhR was increased with aging in the human post-mortem hippocampal samples. The increase was more evident in non-neuronal cells than neurons, especially the cytoplasm of astrocytes displayed a robust immunostaining. They also revealed that there was a strong increase in the expression level of AhR protein in the hippocampal samples of patients with Alzheimer’s disease (AD) as compared to that of healthy elderly people. Currently, the role of AhR signaling in the pathogenesis of AD is unknown. However, Duan et al. [171] demonstrated that the neurotoxicity of β-amyloid peptide was dependent on IDO1/KYN/AhR signaling in rat primary neurons. There is clear evidence that uremic toxins aggravate vascular inflammation in many chronic inflammatory diseases, e.g., in cardiovascular diseases [172]. Uremic toxins, e.g., indoxyl sulfate, derived from the gut microbiota, are potent agonists for AhR factor [173]. Chronic kidney disease (CKD) leads to the accumulation of uremic toxins in the circulation. It has been reported that indoxyl sulfate promotes the AhR-mediated production of ROS/RNS compounds which impair the redox balance of endothelial cells and induce vascular inflammation [174]. These toxins not only enhance inflammation in the kidney, but they accelerate vascular or even organismal aging throughout the body [175, 176]. It is known that the presence of CKD exposes the patient to many other chronic diseases, such as cardiovascular diseases [172] and Alzheimer’s disease [177]. Given that AhR signaling is a pleiotropic regulator in inflammatory states, it will provide a great challenge for drug discovery projects.

## Potential molecular mechanisms associated with the AhR-promoted aging process

The AhR is a transcription factor which integrates many upstream signaling pathways to a multitude of downstream functions. Here, the principles of the regulation will be briefly described, mainly focusing on the mechanisms which appear to control the aging process. The signaling mechanisms and functions undertaken by the AhR factor have been described in detail elsewhere [3, 178, 179]. The AhR protein belongs to the family of bHLH/PAS domain factors possessing functional sites for ligand binding, dimerization, HSP90 interface, and DNA-binding [3]. A cytoplasmic complex of AhR contains the interaction with chaperones p23, XAP2, and HSP90 [180]. After ligand binding, AhR factor is translocated to the nucleus where it heterodimerizes with AhR nuclear translocator (ARNT) protein [181]. The AhR/ARNT complex binds to the specific dioxin/xenobiotic response element (DRE/XRE) and this can activate the transcription of multiple genes. However, AhR factor can also form complexes with other transcription factors, e.g., RelB, E2F1, and estrogen receptor (ER), and subsequently bind to their specific binding sites, i.e., not that of DRE/XRE. These non-canonical pathways indicate that AhR factor can affect gene expression which is not controlled by the AhR/ARNT complex [182]. The ARNT factor can form a complex with the AhR repressor (AhRR) protein which means that AhRR protein competes with AhR factor for binding to the ARNT factor. Given that the AhRR/ARNT complex lacks a transactivation domain, it indicates that the complex inhibits AhR signaling [183]. In addition to the genomic regulation, AhR factor is also able to affect signaling in a non-genomic manner. There is clear evidence that ligand binding to AhR factor in the cytoplasm can activate Src kinase which subsequently stimulates focal adhesion kinase (FAK) and promotes integrin clustering and cellular plasticity in human HepG2 cells [119] (Fig. 2). The AhR/Src signaling can also induce the Src/IDO1 and Src/STAT3 pathways which are inducers of many immunosuppressive properties [154].

Given that AhR factor is a ligand-dependent transcription factor, its ligands, either agonists or antagonists, have a key role in the regulation of its activity. The AhR factor was first characterized as a sensor for environmental toxins, such as TCDD and PAH compounds. In addition to xenobiotic ligands, it has been revealed that it can bind a number of endogenous ligands, such as many tryptophan metabolites as well as the metabolites of the arachidonic acid and heme pathways [178]. There exist significant differences in the specificity of ligands in the activation of AhR factor concerning ligand structures, tissues, and even animal species. Currently, it has been speculated that the most important ligands related to the aging process and age-related diseases could be metabolites of L-tryptophan degradation [179]. In the host cells, the activation of IDO1/IDO2 and tryptophan 2–3-dioxygenase (TDO) stimulates the kynurenine pathway generating kynurenine (KYN) and kynurenic acid (KYNA) which are potent activators of AhR factor [127, 179]. Interestingly, many inflammatory mediators stimulate the activation of IDO1 and the levels of KYN are significantly elevated with aging [126, 127]. Ultraviolet radiation (UVR) generates 6-formylindolo[3,2-b]carbazole (FICZ) from tryptophan and FICZ is a potential inducer of skin photoaging [58]. Gut microbiota are also an important producer of tryptophan metabolites, especially indole compounds, which can gain access to the circulation and subsequently activate AhR signaling in many tissues [173]. Moreover, Marinelli et al. [184] revealed that the butyrate produced by the microbiota was an activating ligand for the AhR factor. There is convincing evidence indicating that microbiome can have a crucial role in the aging process and especially in age-related diseases [185, 186]. Currently, it seems that the IDO1/KYN pathway and gut microbiome provide important endogenous ligands which can participate in the regulation of AhR signaling.

The ARNT protein is not a specific binding partner for AhR and AhRR, but it can also bind some other transcription factors, e.g., hypoxia-inducible factor-1α (HIF-1α) [187]. This means that there exists a competition between the AhR and HIF-1α factors for the recruitment of the ARNT protein and subsequent DNA binding [131, 187, 188]. The ARNT factor has also been called HIF-β because it dimerizes with HIF-1α protein. There is robust evidence that hypoxia/HIF-1α suppressed the gene expression via AhR signaling, whereas the activation of AhR inhibited the HIF-1α-driven gene expression [187–189]. The conditional knockout of mouse *Arnt* gene prevented both the AhR and HIF-1α-driven induction of target genes [190]. It has been demonstrated that an increased expression of HIF-1α and hypoxia resistance are both associated with an extension of lifespan, e.g., in subterranean naked mole-rats [191]. Studies on *C. elegans* have also revealed that hypoxia and the consequent stabilization of HIF-1α protein can extend lifespan in the co-operation with other longevity factors [192]. Currently, it is not known whether an increased HIF-1α stabilization in hypoxic conditions decreases AhR signaling and thus could reduce the AhR-promoted degenerative processes.

There is clear evidence that AhR factor co-operates with the NF-κB signaling system [179]. The NF-κB pathway is a major regulator of immune responses, but it also controls apoptosis, cellular senescence, and even the aging process [193, 194]. Vogel et al. [195] demonstrated that AhR factor interacted with RelB component, a member of NF-κB family driving non-canonical signaling, and subsequently the complex became bound to the specific RelB/AhR responsive element (RelBAhRE) in the promoter of the *IL-8* gene and increased the promoter activity in human macrophages. Interestingly, the RelB/AhR complex was able to bind to specific NF-κB sites as well as to the DRE/XRE sites. The RelB-induced DNA-binding did not require ARNT protein, but protein kinase A (PKA) signaling clearly activated the transcription. Consequently, Ishihara et al. [196] revealed that the RelB-enhanced expression of AhR-driven cytokine genes was dependent on which ligand was binding to the AhR factor. For instance, TCDD and FICZ promoted the RelB-dependent expression of CCL20, whereas indole-3-carbinol suppressed the expression of CCL20 in the LPS-stimulated mouse macrophages. The function of the RelB factor has been associated with many developmental processes in the immune system, especially the differentiation of dendritic cells [197]. It is known that RelB protein can modify the chromatin landscape and thus promote alterations in cell phenotypes. For instance, RelB protein can form a repressive complex with histone H3 lysine methyltransferase G9a in the IL-1β promoter of human THP-1 monocytes and thus induce tolerance to endotoxin [198]. Currently, it is not known whether the RelB/AhR complex can be associated with the RelB-dependent epigenetic regulation.

Currently, it is known that epigenetic regulation controls the expression of the *AhR* gene and subsequently AhR protein can modify, e.g., the activity of retrotransposons [13, 26]. Epigenetic mechanisms have a crucial role both in the early development and the aging process [199, 200]. There are several studies indicating that the promoter sequences of the human *AhR* gene contained DNA methylation sites which controlled the silencing of the gene [201]. Moreover, Ko et al. [24] demonstrated that the promoter of the mouse *AhR* gene contained clusters of binding sites for the pluripotency factors NANOG and OCT3/4 which mediated the silencing of the *AhR* gene through an association of polycomb group (PcG) proteins with the methylated histones. Accordingly, the differentiation of mouse embryonal stem cells increased the acetylation level of these histone sites that enhanced the transcription of the *AhR* gene. Garrison et al. [202] reported that exposure of human HepG2 and MCF7 cells to histone deacetylase (HDAC) inhibitors robustly increased the activity of the *AhR* promoter. These observations highlight that the transactivation of the *AhR* gene is under the epigenetic regulation through DNA and histone methylation. Interestingly, there are studies indicating that AhR factor is able to regulate the epigenetic state of target genes. Singh et al. [203] demonstrated that in mice, an experimental colitis decreased the presence of Tregs and increased the activation of Th17 cells. Interestingly, TCDD exposure promoted the differentiation of Tregs and inhibited the activity of Th17 cells. They also revealed that TCDD treatment significantly reduced the methylation of CpG sites in the promoter of the *FoxP3* gene, whereas it increased the methylation level of the *IL-17* promoter. These changes attenuated clinical and inflammatory markers of mouse colitis. Clinical studies have also revealed that the activation of the AhR-HDAC8 axis promoted the progress of human hepatocellular carcinoma [204]. AhR factor stimulated the expression of HDAC8 via the AhR-ARNT complex in human hepatoma cells. Wajda et al. [205] have reviewed the studies on the role of AhR factor in the epigenetic regulation of the immune system. Currently, it is clear that epigenetic mechanisms control the aging process both at the stem cell and organismal level [96, 200]. Epigenetic regulation is a tempting model to explain how antagonistic pleiotropy could control both the developmental and aging processes.

Several epigenome-wide association studies (EWAS) have demonstrated that smoking is associated with DNA hypomethylation at the intron 3 of the *AhRR* gene (cg05575921) in peripheral blood mononuclear cells (PBMC) [206, 207]. Dawes et al. [208] reported that increased cigarette consumption enhanced the hypomethylation of the intron in PBMC and saliva samples in a dose-dependent manner. Interestingly, there are several reports indicating that the alterations in the DNA methylation signatures of smokers significantly correlated with the markers of the epigenetic aging clock, e.g., with the GrimAge and DNAmPhenoAge biomarkers [209, 210]. These studies indicated that the hypomethylation of CpG site at the intron 3 of the *AhRR* gene accelerated the aging process although the mechanism is still unknown. Moreover, AhR factor is able to regulate the differentiation of embryonic stem cells via the control of retrotransposon *Alu* and *LINE-1* sequences. Alu and LINE-1 transposons are normally silenced with heterochromatin structures, but there are studies indicating that many transposons are activated as the individual ages [211]. Wood et al. [212] demonstrated that the genetic suppression of transposable elements extended the lifespan in *Drosophila*. There are many observations that AhR factor regulates the activity of transposable elements, e.g., Alu and LINE-1 [26, 213] (Fig. 2). It is known that AhR ligands can reactivate LINE-1 retrotransposon in many human and mouse cell lines [213]. The retrotransposition of LINE-1 has been associated with the activation of immune system and thus it can induce both inflammation and autoimmunity [214]. There are observations that LINE-1 caused DNA damages and enhanced cellular senescence [215]. Given that retrotransposons have beneficial effects during embryogenesis, St. Laurent 3^rd^ et al. [215] proposed that the activation of retrotransposition with aging represents an antagonistic pleiotropy in the regulation of the aging process.

## Anti-aging therapeutic treatments suppress AhR signaling

Substantial research efforts have been exerted in the search for anti-aging therapeutics. It is known that metformin and rapamycin treatments are able to extend the healthspan and lifespan of mice [216, 217]. Interestingly, there is clear evidence that metformin and rapamycin can suppress AhR signaling in different models [218, 219]. It seems that autophagy is involved in these results since both metformin and rapamycin are potent inducers of autophagy and it is known that autophagy is an important regulator of the aging process [52, 107]. Interestingly, Yang and Chan [109] demonstrated that the AhR protein was degraded via selective autophagy in several human cell lines. Accordingly, chloroquine, an inhibitor of autophagy, significantly increased the protein level of AhR factor. Nguyen et al. [220] reported that the p23 co-chaperone of the AhR complex prevented the degradation of AhR factor. The levels of AhR and ARNT were also robustly reduced in mouse hepatoma cells with a p23 knockdown. Although the mechanism of p23 in the degradation of AhR factor is unknown, it seems that autophagy controls the protein level and the activity of AhR signaling. In addition to metformin and rapamycin, also a short-term nutrient deprivation was able to induce autophagy and down-regulate AhR factor in human HeLa cells [109]. It is widely accepted that the anti-aging response of caloric restriction is induced by an increase in cellular autophagy [221]. There is robust evidence that certain plant polyphenols are potent modulators of AhR factor, either as agonists or antagonists [222, 223]. Xue et al. [222] categorized different polyphenols according to their agonistic/antagonistic properties; the most potent agonists were chrysin, baicalein, and quercetin, whereas the most powerful antagonists were kaempferol, resveratrol, luteolin, and curcumin. Especially, resveratrol, a potential anti-aging compound, displays many anti-inflammatory and therapeutic effects in age-related degenerative diseases, e.g., cardiovascular diseases, cancers, osteoporosis, neurodegenerative diseases, and sarcopenia [224]. However, the role of AhR signaling as a potential target of anti-aging therapeutics needs to be clarified.

## AhR knockout mice: Cons for antagonistic pleiotropy theory?

The AhR-null (*AhR*^*−/−*^) mice revealed crucial developmental defects in many tissues, especially in liver, spleen, and cardiovascular system [17, 18, 20]. Moreover, there appeared clear impairments in the immune system, e.g., proliferation of hematopoietic stem cells (HSC) was robustly increased which promoted premature exhaustion of HSCs and thus augmented myeloproliferative disorders [19]. The AhR-null mice displayed such pathological changes as cardiovascular lesions, hepatic fibrosis, and increased tumorigenesis [17, 20]. The mortality of the AhR-null mice was significantly increased both right after birth and later in life [17, 225]. All these alterations are consistent with the theory of antagonistic pleiotropy. Surprisingly, recent studies have revealed that lack of AhR factor is associated with premature aging process characterized by enhanced cellular senescence and increased serum levels of both pro-inflammatory and anti-inflammatory cytokines, such as IL-6, TNF-α, and IL-10 [225, 226]. Nacarino-Palma et al. [226] reported that AhR depletion significantly increased tumor incidence in mouse liver with aging. Moreover, they demonstrated that the deficiency of AhR factor robustly expanded the number of senescent cells in the liver preceding the aging process. For instance, there was a clear overexpression of senescence markers, such as senescence-associated β-galactosidase, p16INK4a, and p21CIP1. An increased cellular senescence was not only a liver-specific process since AhR-deficient embryonic and adult fibroblasts displayed increased cellular senescence in vitro cell cultures. These results indicate that lack of AhR factor accelerates premature aging process and tumorigenesis, robustly present already at the age of 12–15 months [225, 226]. Given that AhR signaling promotes cellular senescence with aging (see above), these observations on premature aging process in the AhR-null mice seem to be contradictory to the antagonistic pleiotropy theory (Fig. 1).

There is substantial evidence that natural killer (NK) cells and cytotoxic CD8^+^ T cells have a crucial role in the immune surveillance of senescent and tumor cells [227–229]. Sagiv et al. [227] revealed that NK cells targeted senescent human fibroblast and killed them by secreting perforin, a pore-forming cytolytic protein present in the granules of NK and CD8^+^ T cells. NK and CD8^+^ T cells also exploit the perforin/granzyme mechanism to induce apoptosis of tumor cells [230]. Consequently, Ovadya et al. [231] reported that there was a robust accumulation of senescent cells in the tissues of perforin-knockout mice which displayed a premature aging process. Interestingly, there is convincing evidence that AhR factor is required for the maintenance of liver-resident NK cells in mice [232]. Zhang et al. [232] demonstrated that there was a robust decline in the number of NK cells in the AhR-null mice. Moreover, AhR deficient mice could not mount an NK cell memory response to hapten challenges. Accordingly, Shin et al. [233] reported that the activation of AhR factor was required for the proper cytolytic activity of NK cells in tumor surveillance, i.e., NK cells from the AhR-null mice possessed an intrinsic defect in tumoricidal activity. These results imply that an impaired surveillance capacity of NK cells could cause an accumulation of senescent and tumor cells in the AhR-null mice. An enhanced cellular senescence of AhR-depleted fibroblasts in cell culture might be attributed to metabolic disturbances caused by the absence of the AhR-driven gene expression, both via genomic and non-genomic pathways. It seems that a lack of AhR factor disturbs normal development and leads to pathological processes rather than represents a real aging process.

## Conclusions

The antagonistic pleiotropy theory is an old evolutionary explanation for the cellular senescence and the aging process. There are rather few genes revealing the clear-cut examples of the antagonistic pleiotropy. Blagosklonny [11] provided the evidence that mTOR was a good example of antagonistic pleiotropy, i.e., it drives developmental processes, its knockout has detrimental effects during embryogenesis, but it promotes many age-related degenerative processes later in life. Interestingly, similar properties have been associated with the expression of AhR factor although the mTOR and AhR factors represent very different types of regulation. The AhR factor is an ancient sensor for diverse environmental ligands, initially its role was thought to be confined to defending the organism from a range of chemical threats. Nowadays, it is recognized that it is also involved in early developmental processes, especially the differentiation of stem cells and immune cells. There is substantial evidence that with aging, AhR factor is involved in several processes promoting cellular senescence and age-related pathological conditions, such as osteoporosis, vascular dysfunction, and the remodeling of the immune system. Accordingly, there is evidence that an increase in AhR signaling occurs in many age-related diseases, such as atherosclerosis, cancers, and rheumatoid arthritis. This pleiotropy poses difficulties for targeting AhR for drug discovery purposes. For instance, many AhR agonists, e.g., phytochemicals and microbiota compounds, prevent inflammation but an increase in the level of AhR signaling can promote different age-related degenerative processes. Nonetheless, treatment with tapinarof, a natural AhR agonist, resolved skin inflammation in patients with psoriasis and atopic dermatitis [234]. Currently, there are also some drug discovery projects aimed to reveal novel antagonistic ligands to inhibit AhR signaling. Treatments with AhR antagonists have yielded promising results in rheumatoid arthritis [235] and in cancers, e.g., melanoma and glioma [236]. Some researchers have claimed that AhR factor is a survival and longevity factor based on the health problems and shorter lifespan of the knockout mice. However, it seems that the knockout technology, even a conditional knockout, is unable to confirm directly whether AhR factor is driving the aging process since as a pleiotropic factor AhR signaling is able to regulate different and sometimes opposing traits in the organism. For instance, there are observations that indoles, an agonist of AhR factor, can extend healthspan, but not maximum lifespan in worms, flies, and mice [57] (Fig. 1). However, indoles from commensal bacteria contains numerous compounds which can be processed to different metabolites with diverse agonist/antagonist activities. It is also known that there exist many species-specific differences in the responses of AhR signaling [237] but their role in antagonistic pleiotropy needs to be clarified.

## Data Availability

Not applicable.
